# Supplementary figures and images for: Anti-tumor necrosis factor-alpha monoclonal antibody suppresses colorectal cancer growth in an orthotopic transplant mouse model
Source: PLoS One. 2023 Mar 30;18(3):e0283822. doi: 10.1371/journal.pone.0283822 (PMC10062630; doi:10.1371/journal.pone.0283822)

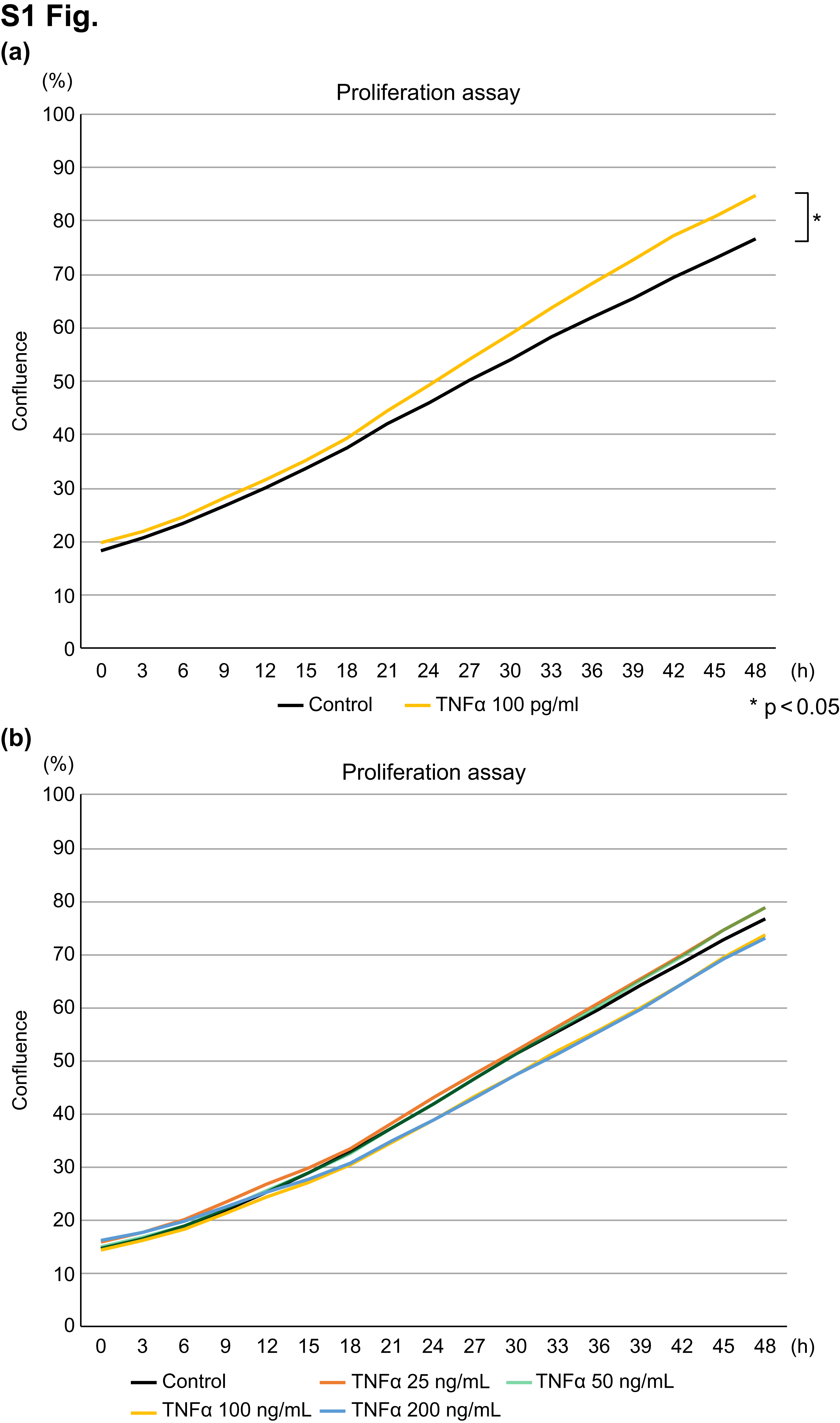

Supplement: S1 Fig — (a) Proliferation ability of CT cells when cultured with TNFα (100 pg/mL). Compared to that in controls, significant cell proliferation was observed at 48 h. Similar results were observed on culture with TNFα at 1, 10, and 1000 pg/mL (data not shown). *P < 0.05. (b) Proliferation ability of CT cells on culture with TNFα (25–200 ng) compared with that of the controls; there was no significant difference with TNFα administration (25, 50, 100, or 200 ng/mL). TNFα, tumor necrosis factor-alpha. (TIF) [file pone.0283822.s001.tif]

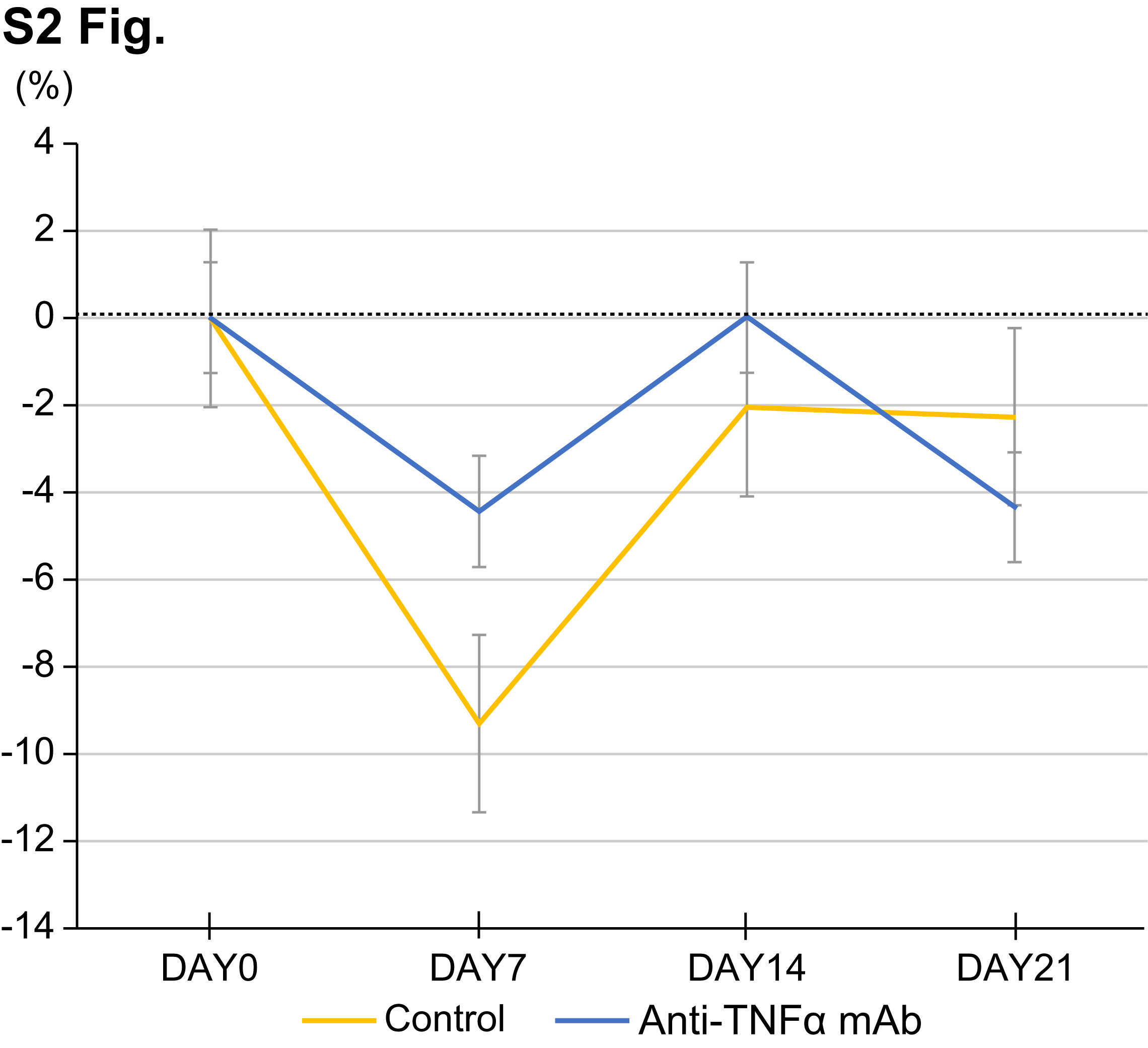

Supplement: S2 Fig — This figure shows the rate of weight change compared to DAY 0. (TIF) [file pone.0283822.s002.tif]
