# Supplementary material for: Anti-tumor necrosis factor-alpha monoclonal antibody suppresses colorectal cancer growth in an orthotopic transplant mouse model
Source: PLoS One. 2023 Mar 30;18(3):e0283822. doi: 10.1371/journal.pone.0283822 (PMC10062630; doi:10.1371/journal.pone.0283822)
Supplement: Attachment — Submitted filename: PONE-D-23-01681_reviewer.pdf [file pone.0283822.s004.pdf]

PLOS ONE

Anti-tumor necrosis factor-alpha monoclonal antibody suppresses colorectal cancer growth in an orthotopic transplant mouse model

--Manuscript Draft--

**Manuscript Number:**	PONE-D-23-01681
**Article Type:**	Research Article
**Full Title:**	Anti-tumor necrosis factor-alpha monoclonal antibody suppresses colorectal cancer growth in an orthotopic transplant mouse model
**Short Title:**	Anti-TNFα mAb suppresses colorectal cancer growth
**Corresponding Author:**	Ryohei Hayashi<br>Hiroshima University Hospital: Hiroshima Daigaku Byoin<br>JAPAN
**Keywords:**	anti-tumor necrosis factor-alpha monoclonal antibody; colorectal cancer; orthotopic transplant mouse model; tumor microenvironment
**Abstract:**	The risk of malignant tumor progression has been a concern associated with the use of anti-tumor necrosis factor-alpha monoclonal antibody (anti-TNFα mAb). On the contrary, recent observational studies have reported negatively on this risk and instead suggested that anti-TNFα mAb acts as a tumor suppressor in inflammatory carcinogenesis models and subcutaneous transplant models of colorectal cancer. However, no consensus has been established regarding the actual effects of anti-TNFα mAb on malignant tumors. Here, we aimed to evaluate, for the first time, the effect of anti-TNFα mAb on the tumor microenvironment in the absence of intestinal inflammation in a colorectal cancer orthotopic transplant mouse model suitable for tumor microenvironment assessment. The orthotopic transplantation model was developed by transplanting CT26 cells into the cecum of BALB/c mice. Changes in tumor size and weight were recorded 3 weeks after transplantation, and the tumor microenvironment was assessed via RNA sequencing and immunohistological staining. In the orthotopic transplant model, the administration of anti-TNFα mAb led to a reduction in colorectal cancer. The RNA sequencing analysis showed upregulation of immune-related pathways and apoptosis and suppression of stromal- and tumor growth-related pathways. Additionally, Gene Ontology analysis showed inhibition of angiogenesis. Immunohistochemical staining showed inhibition of tumor growth, increase in apoptosis, suppression of stromal response, suppression of angiogenesis, enhancement of tumor immunity, and reduction in the number of tumor-associated macrophages. Anti-TNFα mAb acts as an inhibitor of tumor progression in the tumor microenvironment of a colorectal cancer orthotopic transplant mouse model.
**Order of Authors:**	Ryohei Hayashi
	Takeshi Takasago
	Yoshitaka Ueno
	Misa Ariyoshi
	Kana Onishi
	Ken Yamashita
	Yuichi Hiyama
	Hidehiko Takigawa
	Ryo Yuge
	Yuji Urabe
	Shiro Oka
	Yasuhiko Kitadai
	Shinji Tanaka

Powered by Editorial Manager® and ProduXion Manager® from Aries Systems Corporation

Additional Information:	
**Question**	**Response**
**Financial Disclosure**<br><br>Enter a financial disclosure statement that describes the sources of funding for the work included in this submission. Review the submission guidelines for detailed requirements. View published research articles from *PLOS ONE* for specific examples.<br><br>This statement is required for submission and **will appear in the published article** if the submission is accepted. Please make sure it is accurate.<br><br>**Unfunded studies**<br>Enter: *The author(s) received no specific funding for this work.*<br><br>**Funded studies**<br>Enter a statement with the following details:<br>• Initials of the authors who received each award<br>• Grant numbers awarded to each author<br>• The full name of each funder<br>• URL of each funder website<br>• Did the sponsors or funders play any role in the study design, data collection and analysis, decision to publish, or preparation of the manuscript?<br>• **NO** - Include this sentence at the end of your statement: *The funders had no role in study design, data collection and analysis, decision to publish, or preparation of the manuscript.*<br>• **YES** - Specify the role(s) played.<br><br>* typeset	The author received no specific funding for this work
**Competing Interests**<br><br>Use the instructions below to enter a competing interest statement for this submission. On behalf of all authors, disclose any competing interests that could be perceived to bias this work—acknowledging all financial support and any other relevant financial or non-financial competing interests.	The authors have declared that no competing interests exist.

This statement is required for submission and **will appear in the published article** if the submission is accepted. Please make sure it is accurate and that any funding sources listed in your Funding Information later in the submission form are also declared in your Financial Disclosure statement.<br><br>View published research articles from *PLOS ONE* for specific examples.<br><br>**NO authors have competing interests**<br><br>Enter: *The authors have declared that no competing interests exist.*<br><br>**Authors with competing interests**<br><br>Enter competing interest details beginning with this statement:<br><br>*I have read the journal's policy and the authors of this manuscript have the following competing interests: [insert competing interests here]*<br><br>* typeset	
**Ethics Statement**<br><br>Enter an ethics statement for this submission. This statement is required if the study involved:<br><br>• Human participants<br>• Human specimens or tissue<br>• Vertebrate animals or cephalopods<br>• Vertebrate embryos or tissues<br>• Field research<br><br>Write "N/A" if the submission does not require an ethics statement.<br><br>General guidance is provided below. Consult the submission guidelines for detailed instructions. **Make sure that all information entered here is included in the**	Animal experiments in this study were approved by the Animal Experiment Committee of Hiroshima University (A19-144). Anesthesia was induced via an intraperitoneal injection of medetomidine (0.3 mg/kg), midazolam (4 mg/kg), and butorphanol (5 mg/kg)

**Methods section of the manuscript.**<br><br>**Format for specific study types**<br><br>**Human Subject Research (involving human participants and/or tissue)**<br>• Give the name of the institutional review board or ethics committee that approved the study<br>• Include the approval number and/or a statement indicating approval of this research<br>• Indicate the form of consent obtained (written/oral) or the reason that consent was not obtained (e.g. the data were analyzed anonymously)<br><br>**Animal Research (involving vertebrate animals, embryos or tissues)**<br>• Provide the name of the Institutional Animal Care and Use Committee (IACUC) or other relevant ethics board that reviewed the study protocol, and indicate whether they approved this research or granted a formal waiver of ethical approval<br>• Include an approval number if one was obtained<br>• If the study involved *non-human primates*, add *additional details* about animal welfare and steps taken to ameliorate suffering<br>• If anesthesia, euthanasia, or any kind of animal sacrifice is part of the study, include briefly which substances and/or methods were applied<br><br>**Field Research**<br><br>Include the following details if this study involves the collection of plant, animal, or other materials from a natural setting:<br>• Field permit number<br>• Name of the institution or relevant body that granted permission	
**Data Availability**<br><br>Authors are required to make all data underlying the findings described fully available, without restriction, and from the time of publication. PLOS allows rare exceptions to address legal and ethical concerns. See the PLOS Data Policy and FAQ for detailed information.	Yes - all data are fully available without restriction

Powered by Editorial Manager® and ProduXion Manager® from Aries Systems Corporation

A Data Availability Statement describing where the data can be found is required at submission. Your answers to this question constitute the Data Availability Statement and **will be published in the article**, if accepted.<br><br>**Important:** Stating 'data available on request from the author' is not sufficient. If your data are only available upon request, select 'No' for the first question and explain your exceptional situation in the text box.<br><br>Do the authors confirm that all data underlying the findings described in their manuscript are fully available without restriction?	
**Describe where the data may be found in full sentences. If you are copying our sample text, replace any instances of XXX with the appropriate details.**<br><br>• If the data are **held or will be held in a public repository**, include URLs, accession numbers or DOIs. If this information will only be available after acceptance, indicate this by ticking the box below. For example: *All XXX files are available from the XXX database (accession number(s) XXX, XXX.*).<br>• If the data are all contained **within the manuscript and/or Supporting Information files**, enter the following: *All relevant data are within the manuscript and its Supporting Information files.*<br>• If neither of these applies but you are able to provide **details of access elsewhere**, with or without limitations, please do so. For example:<br><br>*Data cannot be shared publicly because of [XXX]. Data are available from the XXX Institutional Data Access / Ethics Committee (contact via XXX) for researchers who meet the criteria for access to confidential data.*<br><br>*The data underlying the results*	The data supporting the findings of this study are available from the corresponding author on reasonable request.

presented in the study are available from (include the name of the third party and contact information or URL).<br>• This text is appropriate if the data are owned by a third party and authors do not have permission to share the data.<br><br>* typeset	
Additional data availability information:	

Powered by Editorial Manager® and ProduXion Manager® from Aries Systems Corporation

Manuscript
Click here to access/download;Manuscript;takasago_Main_text_PLOS_ONE

Anti-tumor necrosis factor-alpha monoclonal antibody suppresses colorectal cancer growth in an orthotopic transplant mouse model

Short title: Anti-TNFα mAb suppresses colorectal cancer growth

Takeshi Takasago[1], Ryohei Hayashi[2*], Yoshitaka Ueno[2], Misa Ariyoshi[1], Kana Onishi[1], Ken Yamashita[2], Yuichi Hiyama[2], Hidehiko Takigawa[2], Ryo Yuge[1], Yuji Urabe[3], Shiro Oka[1], Yasuhiko Kitadai[4], Shinji Tanaka[2]

[1] Department of Gastroenterology, Hiroshima University Hospital, Hiroshima, Japan

[2] Department of Endoscopy, Hiroshima University Hospital, Hiroshima, Japan

[3] Gastroenterointesinal Endoscopy and Medicine, Hiroshima University Hospital, Hiroshima, Japan

[4] Department of Health and Science, Prefectural University of Hiroshima, Hiroshima, Japan

*** Corresponding author**
Email: r-hayashi@hiroshima-u.ac.jp (RH)

Abstract

The risk of malignant tumor progression has been a concern associated with the use of anti-tumor necrosis factor-alpha monoclonal antibody (anti-TNFα mAb). On the contrary, recent observational studies have reported negatively on this risk and instead suggested that anti-TNFα mAb acts as a tumor suppressor in inflammatory carcinogenesis models and subcutaneous transplant models of colorectal cancer. However, no consensus has been established regarding the actual effects of anti-TNFα mAb on malignant tumors. Here, we aimed to evaluate, for the first time, the effect of anti-TNFα mAb on the tumor microenvironment in the absence of intestinal inflammation in a colorectal cancer orthotopic transplant mouse model suitable for tumor microenvironment assessment. The orthotopic transplantation model was developed by transplanting CT26 cells into the cecum of *BALB/c* mice. Changes in tumor size and weight were recorded 3 weeks after transplantation, and the tumor microenvironment was assessed via RNA sequencing and immunohistological staining. In the orthotopic transplant model, the administration of anti-TNFα mAb led to a reduction in colorectal cancer. The RNA sequencing analysis showed upregulation of immune-related pathways and apoptosis and suppression of stromal- and tumor growth-related pathways. Additionally, Gene Ontology analysis showed inhibition of angiogenesis. Immunohistochemical staining showed inhibition of

tumor growth, increase in apoptosis, suppression of stromal response, suppression of angiogenesis, enhancement of tumor immunity, and reduction in the number of tumor-associated macrophages. Anti-TNFα mAb acts as an inhibitor of tumor progression in the tumor microenvironment of a colorectal cancer orthotopic transplant mouse model.

Keywords

anti-tumor necrosis factor-alpha monoclonal antibody, colorectal cancer, orthotopic transplant mouse model, tumor microenvironment

Introduction

Anti-tumor necrosis factor-alpha monoclonal antibody (anti-TNFα mAb) suppresses inflammation by inhibiting the activity of TNFα and is employed as a major therapeutic agent for immune-mediated inflammatory diseases such as inflammatory bowel disease (IBD) and rheumatoid arthritis (RA) [1]. Facilitating the formation of malignant tumors via immunosuppression is a matter of concern [2]. The European Crohn´s and Colitis Organisation (ECCO) guidelines also state that the use of anti-TNFα mAb can be detrimental to patients undergoing cancer treatment and should be avoided for 2 years (5 years for patients with a high risk of recurrence) after cancer treatment [3]. However, varied perspectives have been presented with respect to the actual effect of administering anti-TNFα mAb for the suppression of TNFα in patients with malignant cancers, and no consensus has been established.

Recent observational studies have reported negatively on the increased risk of promotion of malignancy in patients using anti-TNFα mAb [4-6]. In contrast, as sporadic reports of tumor growth during anti-TNFα mAb use have been published [7, 8], the possibility that anti-TNFα mAb use may influence tumor growth cannot be ruled out.

In recent years, anti-TNFα mAb has been increasingly used for the treatment of RA or IBD, such as ulcerative colitis and Crohn’s disease. With an increase in the number of

patients with IBD and RA in higher age groups [9, 10], it is assumed that more patients with carcinoma will be considered for the administration of anti-TNFα mAb. In addition, the administration of infliximab, an anti-TNFα mAb, for immune-related adverse events, such as colitis, associated with the administration of immune checkpoint inhibitors to patients with tumors is expected to increase in the future.

Barring a few studies, no prognostic effect of the administration of anti-TNFα mAb to patients with cancer have been observed in most studies [11]. It is difficult to clinically evaluate the effect of anti-TNFα mAb on tumors alone, as patients with IBD and RA are often administered other immunosuppressive agents. With respect to colorectal cancer (CRC), the effects of anti-TNFα mAb on tumors have been evaluated *in vitro*, in inflammatory carcinogenesis models, and in CRC subcutaneous allogeneic transplant models. The results of these studies have demonstrated possible anti-tumor effects of anti-TNFα mAb [12-14].

However, to the best of our knowledge, there are no reports on the effects of anti-TNFα mAb on orthotopic transplantation of CRC. An orthotopic transplant model reflects the tumor microenvironment (TME) and is recommended for assessing tumor progression, angiogenesis, invasion, stroma, and metastasis [15, 16], whereas subcutaneous xenografts do not reproduce the interactions between the tumor and its microenvironment [17].

Therefore, the orthotopic transplant model is a more suitable experimental system for evaluating TME than the subcutaneous xenograft model. Regarding the inflammatory carcinogenesis model, anti-TNFα mAb may suppress the growth of tumors by inhibiting intestinal inflammation [13, 14]. However, the actual effects of anti-TNFα mAb on tumors have not been fully evaluated owing to the substantial effect of inflammation.

In this study, we aimed to investigate the effects of anti-TNFα mAb on tumor progression and TME in the absence of intestinal inflammation using an allogeneic immune response orthotopic transplantation model of CT26, a CRC cell line derived from *BALB/c* mice.

Materials and Methods

CRC cell lines and culture conditions

CT26 (American Type Culture Collection, Manassas, VA, USA), a clonal cell line derived from colon cancer cells of *BALB/c* mouse, was used as the representative of the CRC cell line.

We cultured CT26 cells in Dulbecco's modified Eagle medium (DMEM; Sigma-Aldrich; Merck KGaA, Germany) at 37°C. DMEM was supplemented with 10% fetal bovine serum (FBS; Sigma-Aldrich, Merck KGaA) and a penicillin–streptomycin mixture. The cells were not cultured for more than 12 weeks after their recovery from the frozen stock.

Evaluation of the effect of TNFα on cell proliferation *in vitro*

CT26 cells (1.0×10^4 cells/well) were seeded and cultured in 24-well plates (ImageLock; Essen Bioscience) containing DMEM supplemented with 0.5% FBS. Recombinant mouse TNF-α (cat. no. 575202; Biolegend, San Diego, CA, USA) was added to DMEM to reach the target concentration. Bright-field images obtained using IncuCyte® Zoom (Essen Bioscience) and IncuCyte software (version 2015A Rev1; Essen Bioscience) automatically expressed cell confluence as a percentage over 2 days to generate a figure of cell proliferation. These experiments were performed in triplicate.

Orthotopic transplantation of tumor cells into experimental animals

Female *BALB/c* mice were obtained from Charles River Japan (Tokyo, Japan) and raised under specific pathogen-free conditions until they reached 8 weeks of age. Animal experiments in this study were approved by the Animal Experiment Committee of Hiroshima University (A19-144). Anesthesia was induced via an intraperitoneal injection of medetomidine (0.3 mg/kg), midazolam (4 mg/kg), and butorphanol (5 mg/kg). Transplantation was performed under a zoom stereomicroscope (Carl Zeiss, Oberkochen, Germany) by injecting 1×10^4 CT26 cells in 30 μL of Hank’s balanced salt solution into the cecal wall of female *BALB/c* mice under anesthesia.

Intraperitoneal administration of anti-TNFα mAb to mice

Anti-TNFα mAb (MP6-XT22, 1 mg/mouse; Biolegend, San Diego, USA) was administered to mice according to a previous study [14].

Mice subjected to orthotopic transplantation were intraperitoneally administered 1 mg of anti-TNFα mAb (MP6-XT22; BioLegend) every 7 days, as shown in Fig 1a (anti-TNFα mAb group, n = 8).

Fig 1. Anti-TNFα mAb suppresses colorectal cancer growth in an orthotopic transplant model. (a) Schematic overview of the orthotopic transplant model of colorectal cancer and anti-TNFα mAb administration. (b) Mice subjected to orthotopic transplantation were administered CT26 cells into the cecum with and without anti-TNFα mAb. (c) The weight and volume of the tumor orthotopically transplanted into the cecum were recorded. Control group (n = 12), anti-TNFα mAb group (n = 8). anti-TNFα mAb, anti-tumor necrosis factor-alpha monoclonal antibody.

Phosphate-buffered saline (500 μL) was administered intraperitoneally to the mice in the control group with the same dosing schedule as that employed for mice in the anti-TNFα mAb group (control group, n = 12). All mice were then analyzed on day 21.

Necropsy procedures

At the time of analysis, the weight of the mice was recorded. After grossly confirming the presence of metastases in the liver, lungs, and lymph nodes, the tumors were excised, and their weight and length were measured. The volume of the tumor was calculated with the following formula: $V = 1/2\ (\text{length} \times \text{width}^2)$. The resected tumors were fixed using a formalin-free immunohistochemistry zinc fixative (BD Pharmingen; BD Biosciences) for 24 h at room temperature. The tumors were then embedded in paraffin for immunohistochemical analysis.

Immunohistological staining

In this experiment, the following primary antibodies were used: monoclonal rabbit anti-Ki67 (dilution, 1:300; cat. no. GTX16667; Genetex, Inc. Irvine, CA, USA), monoclonal rabbit anti-F4/80 (dilution, 1:500; cat. no. D2S9R; Cell Signaling Technology, Inc., Danvers, MA, USA), monoclonal rabbit anti-CD163 (dilution, 1:500; cat. no. ab182422; Abcam, plc., Cambridge, UK), polyclonal rabbit anti-CD31 (dilution, 1:300; cat. no. ab28364; Abcam, plc.), monoclonal rabbit anti-CD8α (dilution, 1:400; cat. no. D4W2Z; Cell Signaling Technology, Inc.), monoclonal rabbit anti-CD4 (dilution, 1:400; cat. no. D7D2Z; Cell Signaling Technology, Inc.), polyclonal goat anti-mouse NKp46/NCR1 (dilution, 10 μg/mL; cat. no. AF2225; R&D Systems, Inc., Minneapolis, MN, USA),

rabbit anti-alpha smooth muscle actin (α-SMA; dilution, 1:50; cat. no. ab5694; Abcam, plc.), polyclonal rabbit anti-mouse type I collagen (dilution, 1:500; cat. no. 20151; Novotec, Bron, France), and polyclonal goat anti-fibronectin (FN; dilution, 1:200; cat. no. sc-6952; Santa Cruz Biotechnology, Inc., Dallas, TX, USA). The following two secondary antibodies were used: polyclonal goat anti-rabbit immunoglobulin/biotinylated antibody (dilution, 1:500; cat. no. E0432; Dako, Glostrup Kommune, Denmark) and polyclonal rabbit anti-goat immunoglobulin/biotinylated antibody (dilution, 1:500; cat. no. E0466, Dako). Apoptosis was quantified using the terminal deoxynucleotidyl transferase dUTP nick-end labeling (TUNEL) assay with the ApopTag Plus Apoptosis Detection kit (#S7101; Millipore-Chemicon International, Temecula, CA).

The fixed tumor tissue was cut into sections of 4 μm thickness and used for immunostaining. Immunostaining was performed to examine CD8, CD4, NKp46/NCR1, αSMA, type 1 collagen, fibronectin, CD31, F4/80, and CD163 according to a previously reported protocol [18]. Ki67 labeling and apoptosis indices were evaluated with reference to a previous report [19]; these indices were defined as the ratio of positive cells to the total number of cells counted; they are expressed as percentage.

All images were captured using an all-in-one fluorescence microscope (BZ-X710, KEYENCE, Osaka, Japan).

Quantification was randomly performed in five different microscopic fields of view (magnification 200×), and NKp46/NCR1 and CD4 were examined at 400× magnification. Positive cell counts, positive areas, and the total number of cells were automatically evaluated using a hybrid cell count application (BZ-H3C; KEYENCE, Osaka, Japan) with BZ-X Analyzer software (BZ-H3A; KEYENCE).

RNA sequencing (RNA-Seq) analysis

Tumors (control group and anti-TNFα mAb group) that developed post-orthotopic transplantation were mechanically isolated using a homogenizer. RNA extraction was then performed using the RNeasy Mini kit (Qiagen, Hilden, Germany) according to the manufacturer's instructions. Library construction and data processing were performed by Qiagen (Hilden, Germany). Functional analysis was performed using ingenuity pathway analysis (IPA) [20]. These data were obtained by Rhelixa (Tokyo, Japan).

Statistical analysis

Significant differences in tumor weight and volume and the immunostaining results of the control and anti-TNFα mAb groups were assessed using Mann–Whitney U test. Differences were considered statistically significant at $P < 0.05$.

Results

Administration of anti-TNFα mAb reduced the size of CT26 tumor in mice

Mice were subjected to orthotopic transplantation, and 1 mg of anti-TNFα mAb was administered intraperitoneally every 7 days as shown in Fig 1a. In the anti-TNFα mAb group, a macroscopic reduction in tumor size was observed compared with that in the control group, along with a significant reduction in tumor weight and volume (Fig 1b, c). No apparent lymph node or liver metastases were observed in both groups. No significant difference was observed in the body weight of mice duringat analysis (Table 1).

Table 1

Results of experiments performed with mice orthotopically transplanted with colorectal cancer cells and treated with anti-TNFα mAb

Group	No.	Body weight, g (range)	Tumor weight, g (range)	Tumor volume, mm^3 (range)	Lymph node metastasis, n	Liver metastasis, n
Control	12	20.0 (16.0–25.0)	0.55 (0.29–0.94)**	250.5 (125–650)*	0/12	0/12
Anti-TNFα mAb	8	20.5 (16.0–23.0)	0.27 (0.08–0.71)	125.0 (62.5–405)	0/8	0/8

Median (range), anti-tumor necrosis factor-alpha monoclonal antibody (anti-TNFα mAb),

*$P < 0.05$ vs. anti-TNFα mAb group, **$P < 0.01$ vs. anti-TNFα mAb group

Immune response was enhanced, and stromal reaction, angiogenesis, and cell proliferation were suppressed

To investigate the effect of anti-TNFα mAb on gene expression in transplanted tumors, mRNA levels were analyzed, and GO and IPA analyses were used to comprehensively examine the differences in gene expression and pathways. The GO analysis showed that GO terms such as "extracellular matrix organization," "positive regulation of cell migration," "angiogenesis," and "epithelial to mesenchymal transition" were significantly downregulated in the group treated with anti-TNFα mAb compared with those in the control group (Fig 2a). The GO terms related to immune response, such as "T cells" and "interferons," were significantly enriched in the group treated with anti-TNFα mAb compared with those in the control group (Fig 2b).

Fig 2. Summary of RNA sequencing analysis of the anti-TNFα mAb group and comparison with the control group. (a) GO terms that were downregulated in the anti-TNFα mAb group compared with those in the control group. GO terms, such as "extracellular matrix organization," "positive regulation of cell migration," "angiogenesis," and "epithelial to mesenchymal transition," were significantly downregulated. Significant changes were defined with adjusted *P*-values of less than 0.05.

Control group (n = 3), anti-TNFα mAb group (n = 3). (b) GO terms that were enriched in the anti-TNFα mAb group compared with those in the control group. GO terms related to immune response, such as "T cells" and "interferons," were significantly enriched. Significant changes were defined with adjusted *P*-values of less than 0.05. Control group (n = 3), anti-TNFα mAb group (n = 3). (c) Canonical pathways identified using IPA that were upregulated or downregulated in the anti-TNFα mAb group compared with those in the control group. Extracted pathways are shown on the horizontal axis (threshold set at *P*-value < 0.05). Pathways with positive z-scores (indicated in orange) were predicted to increase the activity, whereas those with negative z-scores (indicated in blue) were predicted to decrease the activity. Pathways were classified into three categories according to their functions: immune response, stromal reaction, and cell proliferation. The immune responses were predicted to increase the activity, and stromal reaction and cell proliferation were predicted to decrease the activity. Control group (n = 3), anti-TNFα mAb group (n = 3). (d) Network of regulator effects in the anti-TNFα mAb group compared with those in the control group observed using IPA. The main figure presents the predicted functions and diseases that are affected by genes incorporated in the dataset. The terms "migration of cells," "invasive tumor," and "advanced malignant cancer" functions were downregulated, and "apoptosis" was upregulated via suppression of the

expression of genes, such as *MMP9* and *FN1*. Control group (n = 3), anti-TNFα mAb group (n = 3). anti-TNFα mAb, anti-tumor necrosis factor-alpha monoclonal antibody; GO, Gene Ontology; IPA, ingenuity pathway analysis.

The canonical pathways extracted using the IPA predicted the association of the group treated with anti-TNFα mAb with enriched T cell-related pathways and pathways involved in immune response, such as "interferon signaling" and "natural killer cell signaling" compared with those in the control group. In the canonical pathway analysis, pathways related to stromal reaction, such as "tumor microenvironment pathway," "regulation of the epithelial mesenchymal transition by growth factor pathway," "TGF-β signaling," and "inhibition of matrix metalloproteinase" were expected to be suppressed, and pathways related to tumor growth, such as "inhibition of signal transducer and activator of transcription (STAT3) pathway," "IL-6 signaling," "FAT10 cancer signaling pathway," "NF-κB signaling," and "p38 mitogen-activated protein kinase (MAPK) signaling" were expected to be inhibited (Fig 2c). In the main figure depicting regulator effects showed "migration of cells," "invasive tumor," "proliferation of cells," "advanced malignant cancer," and "apoptosis" through the suppression of genes such as *MMP9* and *FN1* (Fig 2d).

These results suggest that the administration of anti-TNFα mAb suppresses tumor-associated pathways. Anti-TNFα mAb may inhibit tumor progression by suppressing cell proliferation, enhancing immunity against tumors, increasing apoptosis, and suppressing stromal reaction and angiogenesis. Based on these results, additional evaluation was performed with immunostaining.

Anti-TNFα mAb suppressed tumor growth and increased apoptosis

To assess the effect of anti-TNFα mAb, orthotopically implanted tumors were analyzed immunohistochemically. The Ki67-labeling index was significantly decreased in the anti-TNFα mAb group, indicating that tumor growth was suppressed by anti-TNFα mAb (Fig 3a, b).

Fig 3. Immunohistochemical analysis for examining cell proliferation and apoptosis in transplanted tumors. (a and b) The number of cells stained positive for Ki67 was reduced in the anti-TNFα mAb group. The number of cells positive for TUNEL increased in the anti-TNFα mAb group. Data are presented as mean ± SEM. $^{**}P < 0.01$. Scale bar, 50 μm. Control group (n = 12), anti-TNFα mAb group (n = 8). TUNEL, terminal deoxynucleotidyl transferase dUTP nick end labeling; anti-TNFα mAb, anti-tumor

necrosis factor-alpha monoclonal antibody.

Apoptosis was evaluated using the TUNEL method. The results showed that apoptosis was enhanced in the anti-TNFα mAb group. This finding is consistent with the upregulation of apoptosis observed in the RNA-Seq analysis (Fig 3a, b).

Anti-TNFα mAb suppressed stromal reaction and angiogenesis

Stromal reaction was evaluated using immunostaining for αSMA, type 1 collagen, and fibronectin. The expression of αSMA, type 1 collagen, and fibronectin was significantly downregulated in the anti-TNFα mAb group, indicating that the stromal reaction was suppressed in this group. These results were consistent with the suppression of stromal reaction observed in the RNA-Seq analysis (Fig 4a, b). Immunostaining of CD31 was also performed to evaluate angiogenesis, and the vascular region was found to be significantly reduced in the anti-TNFα mAb group, indicating that angiogenesis was suppressed in this group. This result was consistent with the inhibition of angiogenesis observed in the RNA-Seq analysis (Fig 4a, b).

Fig 4. Immunohistochemical analysis of transplanted tumors for examination of stromal reaction and angiogenesis. (a and b) The area positive for αSMA, type1

collagen, fibronectin, and CD31 decreased in the anti-TNFα mAb group. Data are presented as mean ± SEM. $^{*}P < 0.05$. $^{**}P < 0.01$. Scale bar, 50 μm. Control group (n = 12), anti-TNFα mAb group (n = 8).

αSMA, α□smooth muscle actin; anti-TNFα mAb, anti-tumor necrosis factor-alpha monoclonal antibody.

Anti-TNFα mAb enhanced tumor immunity and suppressed tumor-associated macrophages

Immunostaining of CD8, CD4, and NKp46/NCR1 (used to evaluate natural killer cells) was performed to assess immunity against the tumor. The number of cells that were positive for the expression of CD8 and NKp46/NCR1 was significantly increased in the anti-TNFα mAb group, indicating that immunity against the tumor was enhanced in the anti-TNFα mAb group. The expression of CD4 did not differ between the groups, which was inconsistent with the RNA-Seq results (Fig 5a, b).

Fig 5. Immunohistochemical analysis of transplanted tumors for immune response and tumor-associated macrophages. (a and b) The number of cells positive for CD8 and NKp46/NCR1 was reduced in the anti-TNFα mAb group. The number of cells positive

for CD4 did not differ between the groups. The expression of F4/80 and CD163 was reduced in the anti-TNFα mAb group. Data are presented as mean ± SEM. * $P < 0.05$. ** $P < 0.01$. Scale bar, 50 μm. Control group (n = 12), anti-TNFα mAb group (n = 8). anti-TNFα mAb group, anti-tumor necrosis factor-alpha monoclonal antibody.

F4/80 (a pan-macrophage marker) and CD163 (an M2 macrophage marker) were used in immunostaining to analyze tumor-associated macrophages [21, 22]. The expression of both F4/80 and CD163 was significantly reduced in the anti-TNFα mAb group, indicating a decrease in the number of TAM (Fig 5a, b).

In summary, the results demonstrated immunohistological changes, such as the suppression of cell proliferation, enhancement of immunity against tumors, enhancement of apoptosis rate, suppression of stromal reaction, and suppression of angiogenesis, in the anti-TNFα mAb group.

Discussion

Studies on CRC have investigated the effects of anti-TNFα mAb on tumors *in vitro* and in CRC subcutaneously implanted models [12-14]. Although the orthotopic model is an excellent model for evaluating the TME [15-17], to the best of our knowledge, no study has examined the effect of anti-TNFα mAb on tumors using this model. This study is the

first to examine the effect of anti-TNFα mAb on the TME using such a CRC orthotopic transplantation model. This enabled us to more comprehensively investigate the TME, including tumor growth, stromal reaction, angiogenesis, TAM, immunity against tumors, and apoptosis, compared to previous studies.

TNFα is a cytokine that was initially identified as a factor responsible for tumor necrosis [23]. Previous studies have suggested that high doses of TNFα administered directly into the tumor induce tumor necrosis. However, in recent years, small doses of TNFα, such as those produced by the tumor, have been considered a possible tumor-promoting agent [24].

Low doses of TNFα promote tumor growth and angiogenesis in mouse models of melanoma, lung cancer, and mammary tumors [25]. In CRC, it has been reported that the actual TNFα concentration in colon cancer tissue is as low as 150 pg/mL [12]. Low concentrations of TNFα are known to enhance the migration and invasion of CRC cells, such as CT26 [26]. In our study, low concentrations of TNFα increased tumor proliferation, similar to the results of the above-mentioned studies. In contrast, higher concentrations of TNFα did not result in an increase in tumor growth or a clear decrease in cell proliferation (S1a, b Fig). These results suggest that TNFα at endogenous tumor levels may aid in the promotion of tumor growth. Indeed, TNFα is a key mediator of

cancer-related inflammation and is involved in tumor progression in the TME [24, 27-29]. Inhibition of TNFα also aids in the suppression of various carcinomas [30-33].

In CRC, high expression of TNFα is a poor prognostic factor. TNFα is highly expressed in *CT26* strains and human CRC, and the anti-tumor effect is enhanced upon administering anti-TNFα mAb in combination with oxaliplatin and 5-fluorouracil [12, 34, 35]. The administration of TNFα for the treatment of CT26 tumors affects tumor cell growth *in vitro* and tumor growth *in vivo* (subcutaneous transplantation) [36]. Therefore, suppressing the activity of TNFα may exert an anti-tumor effect.

In the present study, using an orthotopic transplantation model, we demonstrated that the administration of anti-TNFα mAb inhibits tumor growth, and the underlying mechanisms involves the suppression of stromal response, enhancement of tumor immunity, inhibition of angiogenesis, and increased apoptosis.

Under *in vitro* and *in vivo* (subcutaneous transplantation) conditions, TNFα mediates an increase in tumor growth, and inhibition of the activity of TNFα can lead to a reduction in tumor growth [36, 37]. In line with the previous study results, tumor growth was suppressed by anti-TNFα mAb in the orthotopic transplantation model used in this study. Regarding the mechanism by which TNFα influences tumor growth, it is known that in CRC, one of the receptors of TNFα, namely TNFR2, regulates Ki67 expression, affecting

fibroblast-associated proteins and αSMA, thereby increasing cell growth and migration [38].

In this study, apoptosis was enhanced in the anti-TNFα mAb group. Here, anti-TNFα mAb may have acted as a tumor suppressor. The suppression of NF-κB by anti-TNFα mAb inhibited the activity of antiapoptotic factors and promoted tumor apoptosis in a mouse liver model [27]. In a subcutaneous transplant model of melanoma and lung cancer, the knockout of gene encoding TNF receptor 2, the site of action of TNFα, enhanced apoptosis and suppressed tumor growth [39].

A study performed using colon cancer organoids has shown that TNF-α acts as a major factor mediating endothelial–mesenchymal transition (EMT) in the TME [40, 41]. TNF-α and TGF-β induce EMT via NF-κB in CRC cells, promoting CRC invasion and metastasis [42]. TNF-α is also known to promote TGF-β-induced EMT in human endothelial cells and oral squamous carcinoma-derived cells by enhancing TGF-β signaling. It also promotes cancer-associated fibroblast formation [43].

Matrix metalloproteinases (MMPs) have been implicated in EMT and angiogenesis in tumors [44]. MMP-9, which was found to be suppressed in the RNA-Seq analysis in this study, is known to promote tumor invasion and angiogenesis by activating TGF-β in breast cancer cells [45]. Its expression is induced by tumor-derived TNFα in breast cancer

cells [46]. We speculate that the administration of anti-TNFα mAb may have suppressed the expression of MMP-9 and TGF-β, which may have inhibited angiogenesis and EMT. TNFα induces the downregulation of NCR1/NKp46 in NK cells [47]. In esophageal cancer, TNFα suppresses NK cell function via NF-κB [48]. In melanoma, TNFα inhibition promotes CD8 infiltration into the tumor [49]. As TNF signaling suppresses the functions of CD8 and NK cells, which mediate anti-tumor effects, TNF inhibition may promote their functions, thereby leading to anti-tumor effects. In this study, we observed a few cells positive for CD4 expression; however, no significant difference was observed between the groups. TAMs, which are involved in tumor progression and angiogenesis, were also evaluated immunohistochemically, as TAMs are known to exhibit the M2 phenotype. TNFα acts on tumor growth and macrophage proliferation in CRC cells, and subcutaneous transplantation of CRC cells suppresses the expression of TNFα, resulting in a reduction in the size of tumor and the number of macrophages [37]
The MAPK pathway is one of the pathways involved in differentiation into M2 macrophages, and inhibition of this pathway suppresses the differentiation into M2 macrophages. This mechanism may also inhibit tumor suppression and angiogenesis [50].

In conclusion, anti-TNFα mAb demonstrated the potential to suppress tumor growth,

stromal reaction, and angiogenesis, mediate a reduction in the number of TAMs, increase immunity against tumor and apoptosis, and suppress CRC progression in the TME. This study had some limitations. First, although the short-term effects of anti-TNFα mAb on tumors can be evaluated in basic research, including this study, the long-term effects of anti-TNFα mAb on tumors have not been evaluated. Second, we used only one colon cancer cell line, CT26, and the effects of anti-TNFα mAb on other carcinomas and other colon cancer cell lines were not studied. Therefore, it cannot be concluded that anti-TNFα mAb is effective against other organ cancers and CRC based only on the results of this study. Moreover, it remains unclear whether anti-TNFα mAb is clinically safe in humans. Therefore, additional research on the effects of the use of anti-TNFα mAb on tumors should be conducted to clarify these issues.

Acknowledgments

We would like to thank Editage (www.editage.jp) for English language editing.

A part of this study was performed at the Natural Science Center for Basic Research and Development, Hiroshima University.

Data availability statement

The data supporting the findings of this study are available from the corresponding author on reasonable request.

Funding

The authors received no specific funding for this work.

Competing interests

The authors declare that they have no competing interests.

References

1. Tracey D, Klareskog L, Sasso EH, Salfeld JG, Tak PP. Tumor necrosis factor antagonist mechanisms of action: a comprehensive review. Pharmacol Ther. 2008;117:244-79. PMID: 18155297.

2. Koebel CM, Vermi W, Swann JB, Zerafa N, Rodig SJ, Old LJ, et al. Adaptive immunity maintains occult cancer in an equilibrium state. Nature. 2007;450: 903-907.

3. Annese V, Beaugerie L, Egan L, Biancone L, Bolling C, Brandts C, et al. European Evidence-based Consensus: Inflammatory Bowel Disease and Malignancies. J Crohns Colitis. 2015; 9: 945-965. PMID: 26294789.

4. Muller M, D'Amico F, Bonovas S, Danese S, Peyrin-Biroulet L. TNF Inhibitors and Risk of Malignancy in Patients with Inflammatory Bowel Diseases: A

Systematic Review. J Crohns Colitis. 2021;15: 840-859. PMID: 32915970.

5. Mercer LK, Lunt M, Low AL, Dixon WG, Watson KD, Symmons DP, et al. Risk of solid cancer in patients exposed to anti-tumour necrosis factor therapy: Results from the British Society for Rheumatology Biologics Register for Rheumatoid Arthritis. Ann Rheum Dis. 2015;74: 1087-1093. PMID: 24685910.

6. Waljee AK, Higgins PDR, Jensen CB, Villumsen M, Cohen-Mekelburg SA, Wallace BI, et al. Anti-tumour necrosis factor-α therapy and recurrent or new primary cancers in patients with inflammatory bowel disease, rheumatoid arthritis, or psoriasis and previous cancer in Denmark: A nationwide, population-based cohort study. Lancet Gastroenterol Hepatol. 2020;5: 276-284. PMID: 31836320.

7. do Amaral LCGM, Correa LHL, Accord CC, dos Santos T, Kock BO, Kock KS. Cancer occurrence in patients with inflammatory bowel disease in treatment with anti-TNF: Report of three cases and literature review. Braz J Oncol. 2021;17:e-20200042.

8. Lees CW, Ironside J, Wallace WAH, Satsangi J. Resolution of Non–Small-Cell Lung Cancer after Withdrawal of Anti-TNF therapy. N Eng J Med. 2008; 359: 320-321. PMID: 18635444.

9. Safiri S, Kolahi AA, Hoy D, Smith E, Bettampadi D, Mansournia MA, et al.

Global, regional and national burden of rheumatoid arthritis 1990-2017: A systematic analysis of the Global Burden of Disease study 2017. Ann Rheum Dis. 2019;78: 1463-1471. PMID: 31511227.

10. Stepaniuk P, Bernstein CN, Targownik LE, Singh H. Characterization of inflammatory bowel disease in elderly patients: A review of epidemiology, current practices and outcomes of current management strategies. Can J Gastroenterol Hepatol. 2015;29: 327-333. PMID: 26069892.

11. Phan H, Weideman RA, Cipher DJ, Feagins LA. Safety of tumor necrosis factor inhibitor use in patients with concomitant malignancy. Intest Res. 2020;18: 282-288. PMID: 32252501.

12. Li W, Xu J, Zhao J, Zhang R. Oxaliplatin and Infliximab Combination Synergizes in Inducing Colon Cancer Regression. Med Sci Monit. 2017;23:780-789. PMID: 28190020.

13. Popivanova BK, Kitamura K, Wu Y, Kondo T, Kagaya T, Kaneko S, et al. Blocking TNF-alpha in mice reduces colorectal carcinogenesis associated with chronic colitis. J Clin Invest. 2008;118: 560-570. PMID: 18219394.

14. Onizawa M, Nagaishi T, Kanai T, Nagano K, Oshima S, Nemoto Y, et al. Signaling pathway via TNF-alpha/NF-kappaB in intestinal epithelial cells may be directly

involved in colitis-associated carcinogenesis. Am J Physiol Gastrointest Liver Physiol. 2009;296: G850-859. PMID: 19179628.

15. Fidler IJ. Angiogenic Heterogeneity: Regulation of Neoplastic Angiogenesis by the Organ Microenvironment. JNCI: J Nat Cancer Inst. 2001;93: 1040-1041.

16. Tseng W, Leong X, Engleman E. Orthotopic mouse model of colorectal cancer. J Vis Exp. 2007: 10:484. PMID: 18989400.

17. Oliveira RC, Abrantes AM, Tralhão JG, Botelho MF. The role of mouse models in colorectal cancer research-The need and the importance of the orthotopic models. Animal Model Exp Med. 2020;3: 1-8. PMID: 32318654.

18. Naito T, Yuge R, Kitadai Y, Takigawa H, Higashi Y, Kuwai T, et al. Mesenchymal stem cells induce tumor stroma formation and epithelial□mesenchymal transition through SPARC expression in colorectal cancer. Oncol Rep. 2021;45. PMID: 33907853.

19. Yuge R, Kitadai Y, Shinagawa K, Onoyama M, Tanaka S, Yasui W, et al. mTOR and PDGF pathway blockade inhibits liver metastasis of colorectal cancer by modulating the tumor microenvironment. Am J Pathol. 2015;185: 399-408. PMID: 25478811.

20. Krämer A, Green J, Pollard J, Jr., Tugendreich S. Causal analysis approaches in

Ingenuity Pathway Analysis. Bioinformatics. 2014;30: 523-530. PMID: 24336805.

21. Mantovani A, Sozzani S, Locati M, Allavena P, Sica A. Macrophage polarization: tumor-associated macrophages as a paradigm for polarized M2 mononuclear phagocytes. Trends Immunol. 2002;23: 549-555.. PMID: 12401408.

22. Najafi M, Hashemi Goradel N, Farhood B, Salehi E, Nashtaei MS, Khanlarkhani N, et al. Macrophage polarity in cancer: A review. J Cell Biochem. 2019;120: 2756-2765. PMID: 30270458.

23. Aggarwal BB, Gupta SC, Kim JH. Historical perspectives on tumor necrosis factor and its superfamily: 25 years later, a golden journey. Blood. 2012;119: 651-665. PMID: 22053109.

24. Balkwill F. Tumour necrosis factor and cancer. Nat Rev Cancer. 2009;9: 361-371. PMID: 19343034.

25. Li B, Vincent A, Cates J, Brantley-Sieders DM, Polk DB, Young PP. Low levels of tumor necrosis factor alpha increase tumor growth by inducing an endothelial phenotype of monocytes recruited to the tumor site. Cancer Res. 2009;69: 338-348. PMID: 19118019.

26. Shi G, Zheng X, Zhang S, Wu X, Yu F, Wang Y, et al. Kanglaite inhibits EMT

caused by TNF-α via NF-κB inhibition in colorectal cancer cells. Oncotarget. 2018;9: 6771-6779. PMID: 29467927.

27. Pikarsky E, Porat RM, Stein I, Abramovitch R, Amit S, Kasem S, et al. NF-κB functions as a tumour promoter in inflammation-associated cancer. Nature. 2004; 431: 461-466..

28. Sethi G, Sung B, Aggarwal BB. TNF: A master switch for inflammation to cancer. Front Biosci. 2008;3: 5094-5107.. PMID: 18508572.

29. Balkwill F. TNF-α in promotion and progression of cancer. Cancer and Metastasis Reviews. 2006;25: 409.

30. Charles KA, Kulbe H, Soper R, Escorcio-Correia M, Lawrence T, Schultheis A, et al. The tumor-promoting actions of TNF-alpha involve TNFR1 and IL-17 in ovarian cancer in mice and humans. J Clin Invest. 2009;119: 3011-3023. PMID: 19741298.

31. Cui X, Zhang H, Cao A, Cao L, Hu X. Cytokine TNF-α promotes invasion and metastasis of gastric cancer by down-regulating Pentraxin3. J Cancer. 2020;11: 1800-1807. PMID: 32194791.

32. Wei Q, Singh O, Ekinci C, Gill J, Li M, Mamatjan Y, et al. TNFα secreted by glioma associated macrophages promotes endothelial activation and resistance

against anti-angiogenic therapy. Acta Neuropathol Commun. 2021;9: 67. PMID: 33853689.

33. Zhao X, Fan W, Xu Z, Chen H, He Y, Yang G, et al. Inhibiting tumor necrosis factor-alpha diminishes desmoplasia and inflammation to overcome chemoresistance in pancreatic ductal adenocarcinoma. Oncotarget. 2016;7: 81110-81122. PMID: 27835602.

34. Liu F, Ai F, Tian L, Liu S, Zhao L, Wang X. Infliximab enhances the therapeutic effects of 5-fluorouracil resulting in tumor regression in colon cancer. Onco Targets Ther. 2016;9:5999-6008. PMID: 27757041.

35. Huang D, Xue J, Li S, Yang D. Oxaliplatin and infliximab synergize to induce regression of colon cancer. Oncol Lett. 2018;15: 1517-1522. PMID: 29434844.

36. Ray AL, Berggren KL, Restrepo Cruz S, Gan GN, Beswick EJ. Inhibition of MK2 suppresses IL-1β, IL-6, and TNF-α-dependent colorectal cancer growth. Int J Cancer. 2018;142: 1702-1711. PMID: 29197088.

37. Zins K, Abraham D, Sioud M, Aharinejad S. Colon Cancer Cell–Derived Tumor Necrosis Factor-α Mediates the Tumor Growth–promoting Response in Macrophages by Up-regulating the Colony-Stimulating Factor-1 Pathway. Cancer Res. 2007;67: 1038-1045.

38. Wang L, Yang D, Tian J, Gao A, Shen Y, Ren X, et al. Tumor necrosis factor receptor 2/AKT and ERK signaling pathways contribute to the switch from fibroblasts to CAFs by progranulin in microenvironment of colorectal cancer. Oncotarget. 2017;8: 26323-26333. PMID: 28412748.

39. Sasi SP, Yan X, Enderling H, Park D, Gilbert HY, Curry C, et al. Breaking the 'harmony' of TNF-α signaling for cancer treatment. Oncogene. 2012;31: 4117-4127.

40. Bates RC, Mercurio AM. Tumor necrosis factor-alpha stimulates the epithelial-to-mesenchymal transition of human colonic organoids. Mol Biol Cell. 2003;14: 1790-1800. PMID: 12802055.

41. Wang H, Wang HS, Zhou BH, Li CL, Zhang F, Wang XF, et al. Epithelial-mesenchymal transition (EMT) induced by TNF-α requires AKT/GSK-3β-mediated stabilization of snail in colorectal cancer. PLoS One. 2013;8: e56664. PMID: 23431386.

42. Li Y, Zhu G, Zhai H, Jia J, Yang W, Li X, et al. Simultaneous stimulation with tumor necrosis factor-α and transforming growth factor-β1 induces epithelial-mesenchymal transition in colon cancer cells via the NF-κB pathway. Oncol Lett. 2018;15: 6873-6880. PMID: 29725419.

43. Yoshimatsu Y, Wakabayashi I, Kimuro S, Takahashi N, Takahashi K, Kobayashi M, et al. TNF-α enhances TGF-β-induced endothelial-to-mesenchymal transition via TGF-β signal augmentation. Cancer Sci. 2020;111: 2385-2399. PMID: 32385953.

44. Quintero-Fabián S, Arreola R, Becerril-Villanueva E, Torres-Romero JC, Arana-Argáez V, Lara-Riegos J, et al. Role of Matrix Metalloproteinases in Angiogenesis and Cancer. Front Oncol. 2019;9:1370. PMID: 31921634.

45. Yu Q, Stamenkovic I. Cell surface-localized matrix metalloproteinase-9 proteolytically activates TGF-beta and promotes tumor invasion and angiogenesis. Genes Dev. 2000; 14(2): 163-176. PMID: 10652271.

46. Stuelten CH, DaCosta Byfield S, Arany PR, Karpova TS, Stetler-Stevenson WG, Roberts AB. Breast cancer cells induce stromal fibroblasts to express MMP-9 via secretion of TNF-alpha and TGF-beta. J Cell Sci. 2005;118: 2143-2153. PMID: 15855236.

47. Ivagnès A, Messaoudene M, Stoll G, Routy B, Fluckiger A, Yamazaki T, et al. TNFR2/BIRC3-TRAF1 signaling pathway as a novel NK cell immune checkpoint in cancer. Oncoimmunology. 2018;7: e1386826. PMID: 30524877.

48. Zheng Y, Li Y, Lian J, Yang H, Li F, Zhao S, et al. TNF-α-induced Tim-3

expression marks the dysfunction of infiltrating natural killer cells in human esophageal cancer. J Transl Med. 2019;17: 165.

49. Bertrand F, Rochotte J, Colacios C, Montfort A, Tilkin-Mariamé AF, Touriol C, et al. Blocking Tumor Necrosis Factor α Enhances CD8 T-cell-Dependent Immunity in Experimental Melanoma. Cancer Res. 2015;75: 2619-2628. PMID: 25977337.

50. Neamatallah T. Mitogen-Activated Protein Kinase Pathway: A Critical Regulator in Tumor-associated Macrophage Polarization. J Microsc Ultrastruct. 2019;7: 53-56. PMID: 31293885.

Supplementary Information

S1 Fig. Evaluation of the effect of TNFα on cell proliferation.

Fig. 1

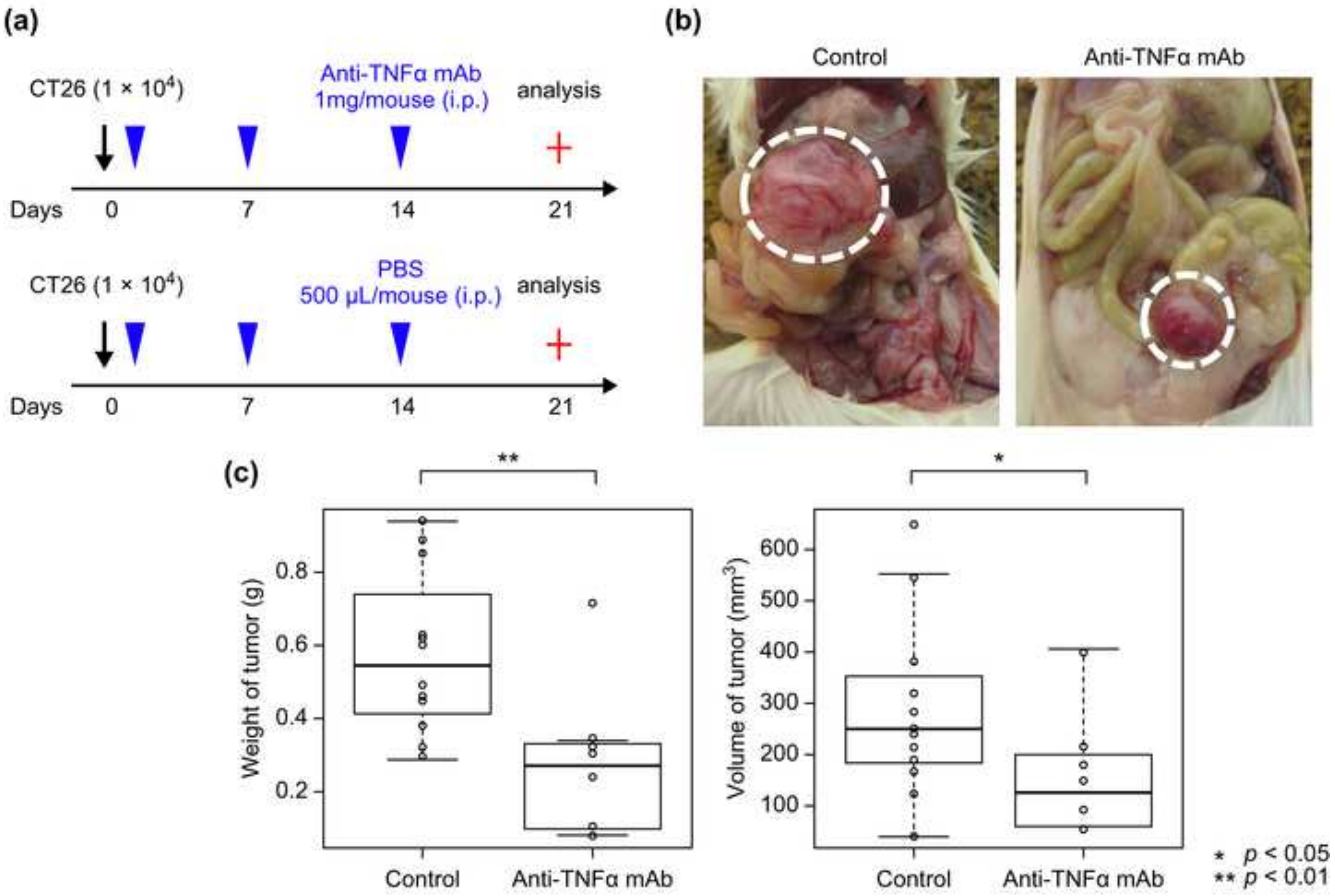
(a)
CT26 (1 × 10⁴)
Anti-TNFα mAb
1mg/mouse (i.p.)
analysis
Days 0 7 14 21
CT26 (1 × 10⁴)
PBS
500 μL/mouse (i.p.)
analysis
Days 0 7 14 21
(b)
Control
Anti-TNFα mAb
(c)
**
*
Weight of tumor (g)
0.2 0.4 0.6 0.8
Volume of tumor (mm³)
100 200 300 400 500 600
Control
Anti-TNFα mAb
Control
Anti-TNFα mAb
* p < 0.05
** p < 0.01

Fig. 2

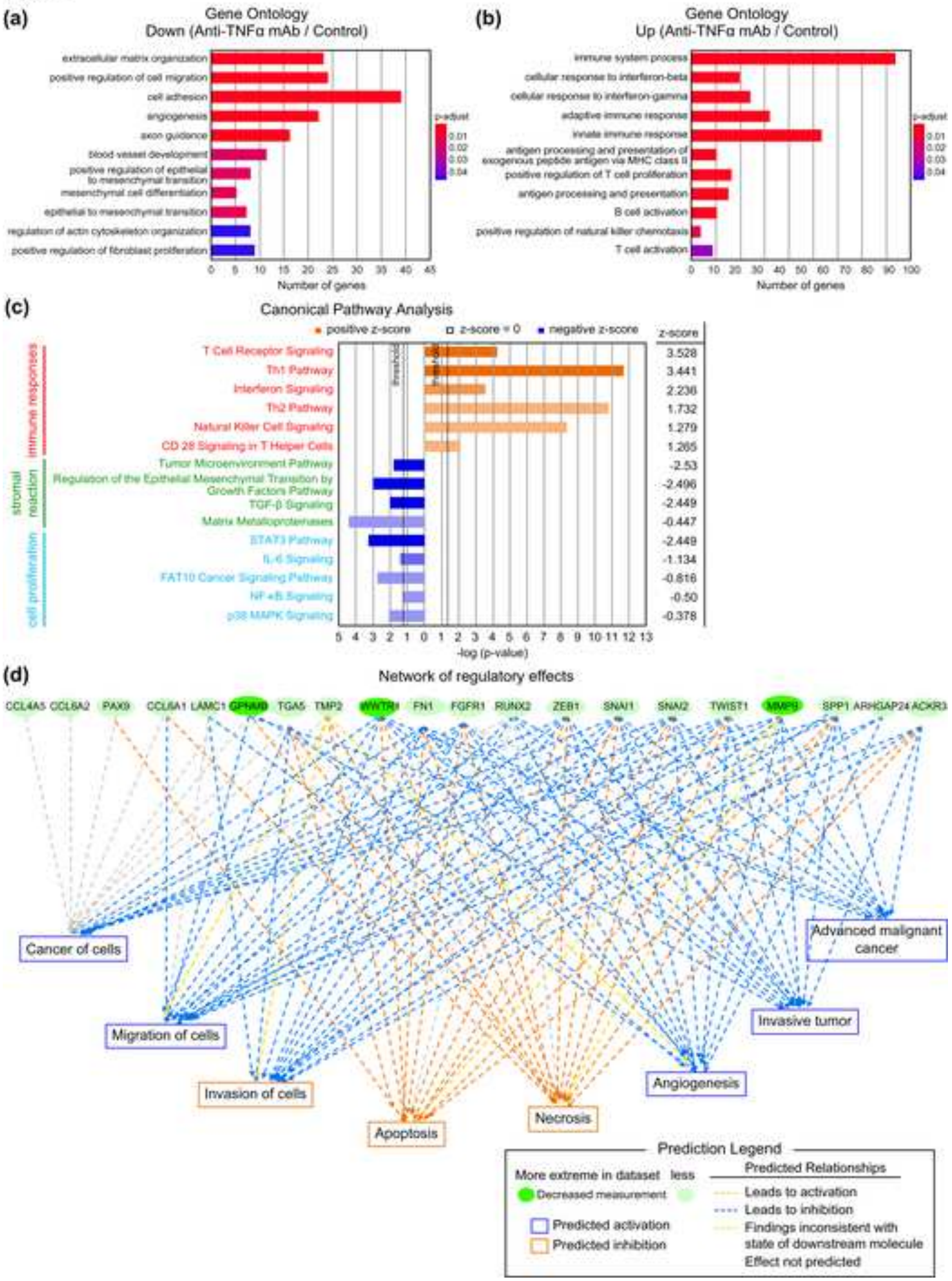
(a)
Gene Ontology
Down (Anti-TNFα mAb / Control)
extracellular matrix organization
positive regulation of cell migration
cell adhesion
angiogenesis
axon guidance
blood vessel development
positive regulation of epithelial to mesenchymal transition
mesenchymal cell differentiation
epithelial to mesenchymal transition
regulation of actin cytoskeleton organization
positive regulation of fibroblast proliferation
Number of genes
p-adjust
(b)
Gene Ontology
Up (Anti-TNFα mAb / Control)
immune system process
cellular response to interferon-beta
cellular response to interferon-gamma
adaptive immune response
innate immune response
antigen processing and presentation of exogenous peptide antigen via MHC class II
positive regulation of T cell proliferation
antigen processing and presentation
B cell activation
positive regulation of natural killer chemotaxis
T cell activation
Number of genes
(c)
Canonical Pathway Analysis
positive z-score
z-score = 0
negative z-score
z-score
immune responses
stromal reaction
cell proliferation
T Cell Receptor Signaling 3.528
Th1 Pathway 3.441
Interferon Signaling 2.236
Th2 Pathway 1.732
Natural Killer Cell Signaling 1.279
CD 28 Signaling in T Helper Cells 1.265
Tumor Microenvironment Pathway -2.53
Regulation of the Epithelial Mesenchymal Transition by Growth Factors Pathway -2.496
TGF-β Signaling -2.449
Matrix Metalloproteinases -0.447
STAT3 Pathway -2.449
IL-6 Signaling -1.134
FAT10 Cancer Signaling Pathway -0.816
NF-κB Signaling -0.50
p38 MAPK Signaling -0.378
threshold
-log (p-value)
(d)
Network of regulatory effects
CCL4A5 CCL6A2 PAX9 CCL6A1 LAMC1 GPNMB TGA5 TMP2 WWTR1 FN1 FGFR1 RUNX2 ZEB1 SNAI1 SNAI2 TWIST1 MMP9 SPP1 ARHGAP24 ACKR3
Cancer of cells
Migration of cells
Invasion of cells
Apoptosis
Necrosis
Angiogenesis
Invasive tumor
Advanced malignant cancer
Prediction Legend
More extreme in dataset less
Decreased measurement
Predicted activation
Predicted inhibition
Predicted Relationships
Leads to activation
Leads to inhibition
Findings inconsistent with state of downstream molecule
Effect not predicted

Click here to access/download;Figure;Fig3.tif

Fig. 3

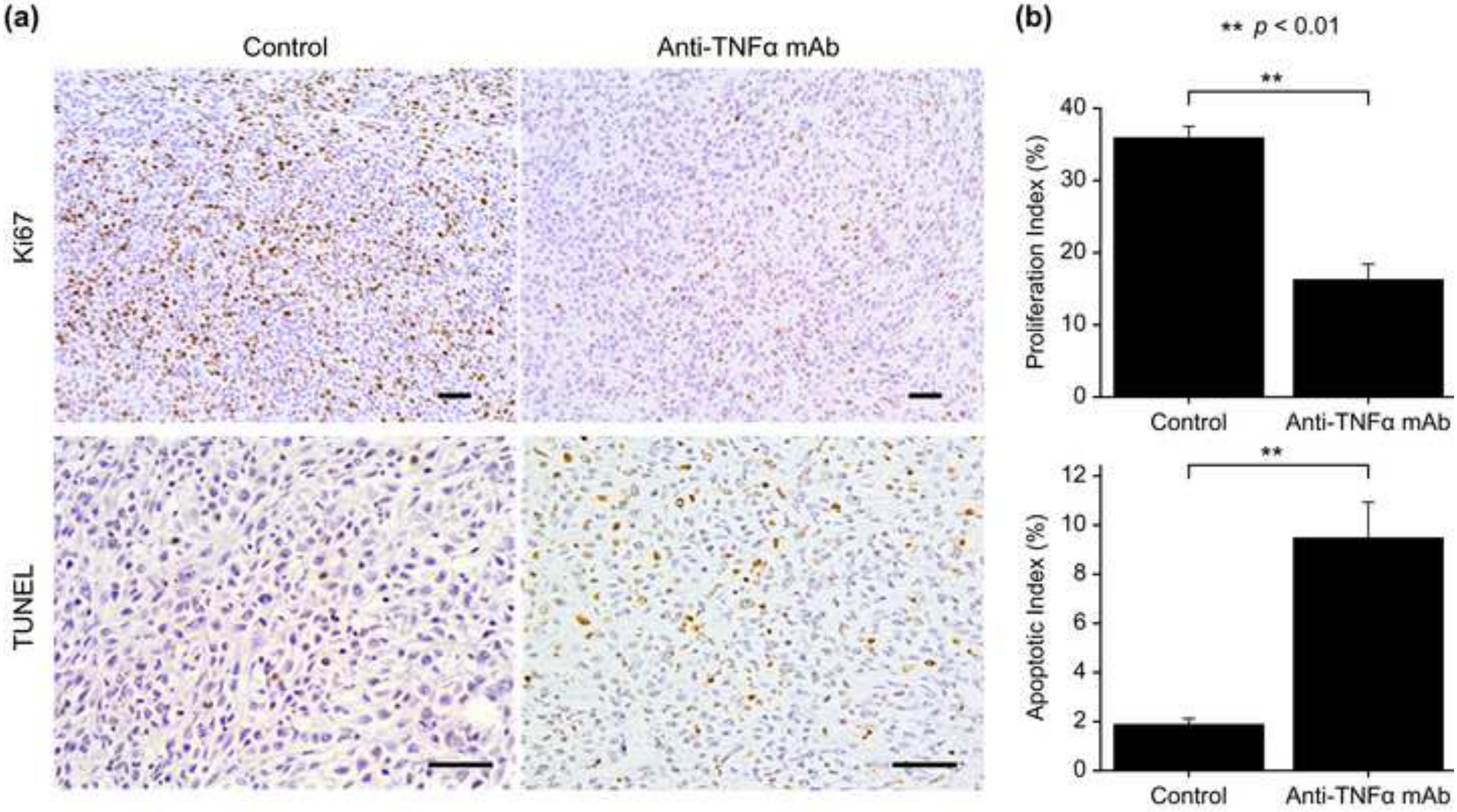

Figure

Click here to access/download;Figure;Fig4.tif

Fig. 4

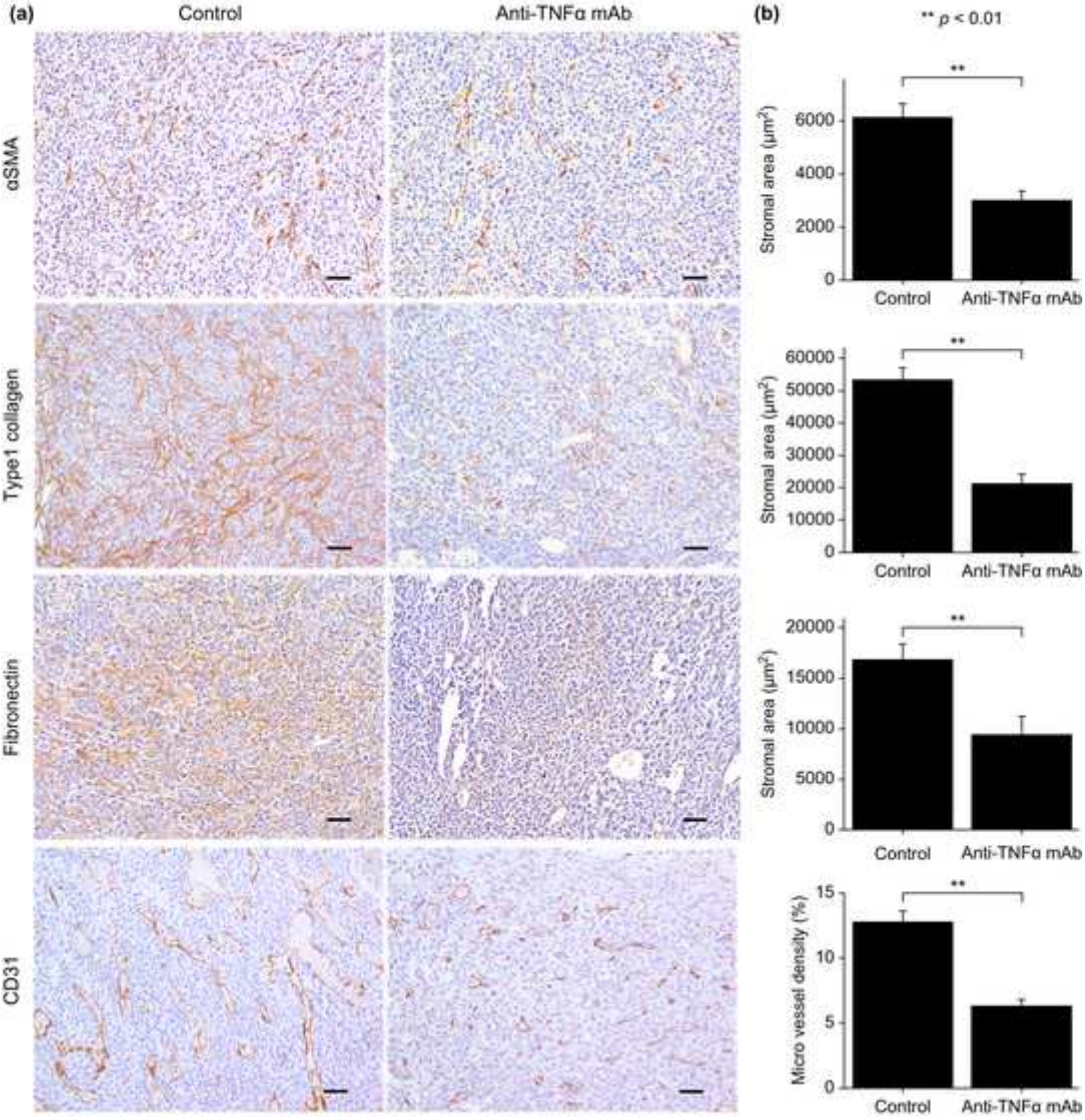

Figure

Click here to access/download;Figure;Fig5.tif

Fig. 5

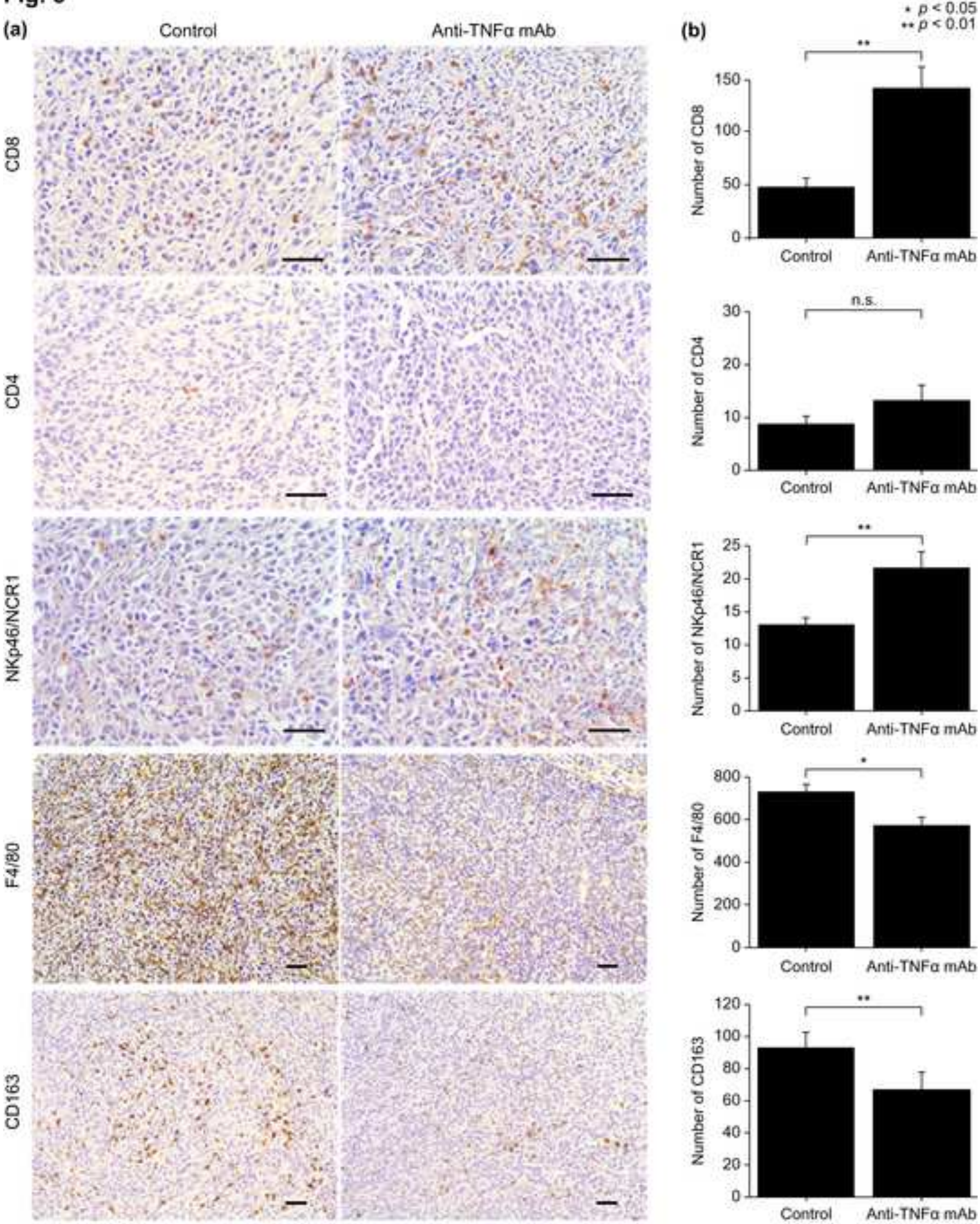

Figure

Click here to access/download
Supporting Information
Sup Fig1.tif

Click here to access/download
Supporting Information
takasago_Supplementary_Material_PLOSONE.docx
