# Supplementary material for: Anti-tumor necrosis factor-alpha monoclonal antibody suppresses colorectal cancer growth in an orthotopic transplant mouse model
Source: PLoS One. 2023 Mar 30;18(3):e0283822. doi: 10.1371/journal.pone.0283822 (PMC10062630; doi:10.1371/journal.pone.0283822)
Supplement: Attachment — Submitted filename: Response to Reviewers.docx [file pone.0283822.s005.docx]

Thank you for your insightful comments. We deeply appreciate the time and effort you have invested in reviewing our manuscript. Below are our responses to each of **your comments and suggestions.** The revisions to the manuscript are indicated in red font (pages and lines are shown in green).

**Reviewer #1: This manuscript provides an interesting investigation into the effects of anti-TNF-a treatment of colorectal cancer in an orthotopic mouse model. However, several concerns with the manuscript need to be addressed.**

**1. Only a single high dose of anti TNF antibody was used (1mg/mouse) both in this manuscript and in the publication cited as a reference for this method. Was a dose response experiment performed in vivo to determine the effective dose of MP6-XT22?**

**➡**Thank you for pointing this out. Accordingly, we have referred to the previous report (Onizawa et al. Am J Physiol Gastrointest Liver Physiol. 2009) and have used the dose in this case. While we believe that a dose reaction experiment is an excellent suggestion, we did not perform a dose reaction experiment here. Experiments have been conducted at similar dose (1 mg/week) in other studies (Jan Sodenkamp et al. Immunobiology. 2012, CA Scanga et al. Infect Immun. 1999) using MP6-XT22. One study experimented with a dose of 0.5 mg × 2/week. In the above study (CA Scanga et al. Infect Immun. 1999), experiments were conducted at 0.5 mg × 2/week and 1 mg/week doses with similar results. Thus, MP6-XT22 (1 mg/week) was considered to be a suitable dose for the experiment.

**2. The method and results sections for RNA sequencing and analysis lacks important details. What methods were used to process sequencing reads, align them to a reference genome, and count the reads aligning to genes? How many genes were differentially expressed in the treatment vs control groups? What statistical method was used to determine whether changes in gene expression were significant?**

**➡**Thank you for pointing this out. We have added the following text to the Methods section regarding sequencing leads and statistical methods; 661 genes were differentially expressed in the treatment vs control groups. (S1 Appendix)

(page 12, line 5–page 13, line 6)

The following library preparation kits and sequencing equipment manufacturer’s protocols were used: for poly(A) RNA preparation, Poly(A) mRNA Magnetic Isolation Module (New England Biolabs, Ipswich, MA, USA); for library preparation, NEBNext® Ultra™II Directional RNA Library Prep Kit for Illumina® (New England Biolabs, Ipswich, MA, USA); for sequencing, NovaSeq 6000 (Illumina Inc., San Diego, CA).

The quality of the raw paired-end sequence reads was assessed with FastQC (Version 0.11.7; https://www.bioinformatics.babraham.ac.uk/projects/fastqc/). Low-quality (< 20) bases and adapter sequences were trimmed using Trimmomatic software (Version 0.38) with the following parameters: ILLUMINACLIP: path/to/adapter.fa:2:30:10 LEADING:20 TRAILING:20 SLIDINGWINDOW:4:15 MINLEN:36. The trimmed reads were aligned to the reference genome using RNA-seq aligner HISAT2 (Version 2.1.0). The HISAT2-resultant .sam files were converted into .bam files using samtools and were used to estimate the abundance of uniquely mapped reads with featureCounts (Version 1.6.3). The raw counts were normalized with transcripts per million (TPM). Based on the normalized read counts, we conducted comparative analyses of samples.

Differentially expressed genes (DEGs) were detected using DESeq2 (Version 1.24.0) with the threshold of |log2FC (Fold Change)| > 1 and p.adjust < 0.05 calculated using the Benjamini and Hochberg (BH) method. Gene set enrichment analysis was conducted using DAVID (Version 1.22.0) and gene ontology (GO) terms with p.adjust < 0.05 by the BH method were extracted. (Fig 2a,2b)

**3. Why was Mann–Whitney U test chosen for all statistical analysis? Were the assumptions of parametric tests violated by skew or unequal variance in the data?**

**➡**The Mann-Whitney U test was chosen because of the non-normal distribution of data.

**4. Body weight is provided at endpoint showing no difference between control and anti-TNF groups but weight at day 0 is not provided. Plotting body weight at multiple time points over the course of the experiment would allow for visualization of any weight loss occurring during tumor growth.**

**➡**Thank you for pointing this out. Accordingly, we have added the day 0 weight (before) to the Table1. Further, the percentage change in weight from DAY 0 is shown in the graph below. (S2 Fig)

**5. Manuscript needs to be edited for spelling and grammar.**

**➡**Thank you for your advice. We have had the manuscript proofread by Editage.

**6. The legend for figure 2 is overly detailed. Some information provided in the fig 2 legend would be more appropriate in the results or methods section.**

**➡**Thank you for pointing this out. As indicated below, the red text is already in the Result section and will be removed from the figure legend. The green text will be moved to the Method section. (page 13, line 10–14)

**Fig 2. Summary of RNA sequencing analysis of the anti-TNFα mAb group and comparison with the control group.** (a) GO terms that were downregulated in the anti-TNFα mAb group compared with those in the control group. ~~GO terms, such as “extracellular matrix organization,” “positive regulation of cell migration,” “angiogenesis,” and “epithelial to mesenchymal transition,” were significantly downregulated.~~ Significant changes were defined with adjusted *P*-values of less than 0.05. Control group (n = 3), anti-TNFα mAb group (n = 3). (b) GO terms that were enriched in the anti-TNFα mAb group compared with those in the control group. ~~GO terms related to immune response, such as “T cells” and “interferons,” were significantly enriched.~~ Significant changes were defined with adjusted *P*-values of less than 0.05. Control group (n = 3), anti-TNFα mAb group (n = 3). (c) Canonical pathways identified using IPA that were upregulated or downregulated in the anti-TNFα mAb group compared with those in the control group. Extracted pathways are shown on the horizontal axis (threshold set at *P*-value < 0.05). Pathways with positive z-scores (indicated in orange) were predicted to increase the activity, whereas those with negative z-scores (indicated in blue) were predicted to decrease the activity. Pathways were classified into three categories according to their functions: immune response, stromal reaction, and cell proliferation. ~~The immune responses were predicted to increase the activity, and stromal reaction and cell proliferation were predicted to decrease the activity.~~ Control group (n = 3), anti-TNFα mAb group (n = 3). (d) Network of regulator effects in the anti-TNFα mAb group compared with those in the control group observed using IPA. ~~The main figure shows functions and diseases predicted by changes in gene expression. The main figure presents the predicted functions and diseases that are affected by genes incorporated in the dataset. The terms “migration of cells,” “invasive tumor,” and “advanced malignant cancer” functions were downregulated, and “apoptosis” was upregulated via suppression of the expression of genes, such as~~ *~~MMP9~~* ~~and~~ *~~FN1~~*~~.~~ Control group (n = 3), anti-TNFα mAb group (n = 3). anti-TNFα mAb, anti-tumor necrosis factor-alpha monoclonal antibody; GO, Gene Ontology; IPA, ingenuity pathway analysis.

**7. Sequencing raw data and/or comprehensive DEG dataset should be uploaded to a repository such as the NCBI Gene Expression Omnibus Database to facilitate access.**

**➡**Thank you for your suggestion. We have uploaded the sequencing data to the NCBI Gene Expression Omnibus. (https://www.ncbi.nlm.nih.gov/geo/query/acc.cgi?acc=GSE226619) Accession number is GSE226619.

**8. The results section describing figure 2 C and D is difficult to understand. Were these pathways “expected to be inhibited or suppressed” or were they found to be inhibited based on analysis of the data? Which specific gene’s expression changes led to the conclusion that each of these pathways are inhibited?**

**➡** In Fig 2c, these are pathways that are expected to be "increased or decreased" on analyzing data on changes in gene expression, relative to known databases. The actual changes in gene expression are presented in the S1 Appendix. (page 13, line 6)

In Fig 2d, functions and diseases predicted to be affected by the gene-expression changes indicated by the green circles are visualized as a network.

As this was unclear in the manuscript text, more details have been added to the Method section (page 13, line 9–17)

**9. Figure 5, the images shown in A do not appear to be accurately represented by the graphs shown in B. This is especially evident in the F4/80 staining where control appears much greater than TNF but the graph only shows a 15% change. Why are the different stainings imaged at different magnifications as evidenced by the change in size of the scale bar?**

**➡**Thank you for your suggestion. Accordingly, we have changed the images to represent the numbers in the graphs accurately and have standardized the magnification to be the same.（Fig3.4,5）

**10. The methods state that “quantification was randomly performed in 5 different fields of view” but the legend for figure 4 states that control had an n of 12 and TNF had an n of 8. It should be clarified whether each n represents an individual mouse, tumor section, or field of view.**

**➡**Thank you for your suggestion. We have revised this to n = 12 (x5 fields) for the control group and n = 8 (x5 fields) for the TNF group. (page 21, line 8–9)

**11. The conclusion rightfully identifies several limitations with the study however these limitations should be discussed in more detail. CT26 is known to have a high mutation rate and be highly immunogenic. It would be interesting to compare the results of the treatment in a more poorly immunogenic tumor such as MC-38 or in a spontaneous model of colon cancer. Furthermore, the results of the present study appear to indicate significant changes in the immune response within the tumor microenvironment however, additional experiments to better characterize these changes would greatly strengthen the impact of the paper such as flow cytometry, immune-phenotyping of T cells and macrophages infiltrating the tumor, or cytokine expression array to quantify cytokine levels within the tumor tissue. As it stands, only very limited conclusions can be drawn from the data presented.**

**➡**As you have pointed out, we believe that it would be interesting to compare our experimental results with those of MC-38, which is less immunogenic than CT26, or with those of spontaneous models of colorectal cancer. We would also be interested in more detailed experiments with flow cytometry, immunophenotyping of tumor-infiltrating T cells and macrophages, or cytokine expression arrays to quantify cytokine levels in tumor tissue.

However, we have not been able to investigate these points in our experiments, and we consider this to be a subject for future research. Accordingly, we have added this point to the limitations section.

(page 27, line 10– page 28, line 1)

Although CT26 is known to have a high mutation rate and high immunogenicity, it is interesting to compare treatment outcomes in less immunogenic tumors such as MC-38 and spontaneous models of colorectal cancer; this is a subject for future study.

Third, this study showed significant changes in immune responses in the tumor microenvironment; however, these findings have not been evaluated in detail, such as by flow cytometry. we believe that these are vital future research topics.

**Reviewer #2: The authors have evaluated anti-tumor efficacy of anti-TNFα mAb against CT26 orthotopic model.**

**The results indicated tumor reduction in anti-TNFα mAb received mice. RNA sequencing analysis showed up-regulation of immune-related pathways and apoptosis and suppression of stromal- and tumor growth-related pathways in the tumor microenvironment (TME).**

**In addition, immunohistochemistry of tumors showed up-regulated infiltration of CD8 cells in TME. TUNEL assay indicated up-regulation of apoptosis in tumors.**

**Tumor-associated macrophages (TAMs) were analyzed using F4/80 and CD163 as markers. The results indicated TAMs suppression in TME.**

**Comments:**

1) **Legends for figures are scattered in the manuscript, please arrange it.**

**➡**Thank you for pointing this out. We have presented the figure legends in accordance with the submission rules. The Fig 1 legend has been moved appropriately. (page 15, line 4–10).

We apologize for any confusion, and thank you for your understanding.

2) **Legend for Fig 1 is missing! Please include it.**

**➡** We apologize for lack of clarity in the placement of Fig 1. This has now been revised. (page 15, line 4–10).

3) **The results of evaluation of the effect of TNFα on cell proliferation is not mentioned in results section.**

➡Thank you for pointing this out. Accordingly, this result has been added to the Result section. (page 14, line 7–13)

4) **The authors showed down-regulation of TAMs in TME. What about M1 macrophages? Did they show up-regulation?**

**➡** We have made several attempts to evaluate M1 macrophages by immunostaining, but faced considerable challenges in completing these evaluations. We apologize for not being able to meet your expectations in this regard.
